# Supplementary material for: In-section Click-iT detection and super-resolution CLEM analysis of nucleolar ultrastructure and replication in plants
Source: Nat Commun. 2024 Mar 19;15:2445. doi: 10.1038/s41467-024-46324-6 (PMC10950858; doi:10.1038/s41467-024-46324-6)
Supplement: Supplementary file 1 — Supplementary Information [file 41467_2024_46324_MOESM1_ESM.pdf]

Supplementary information

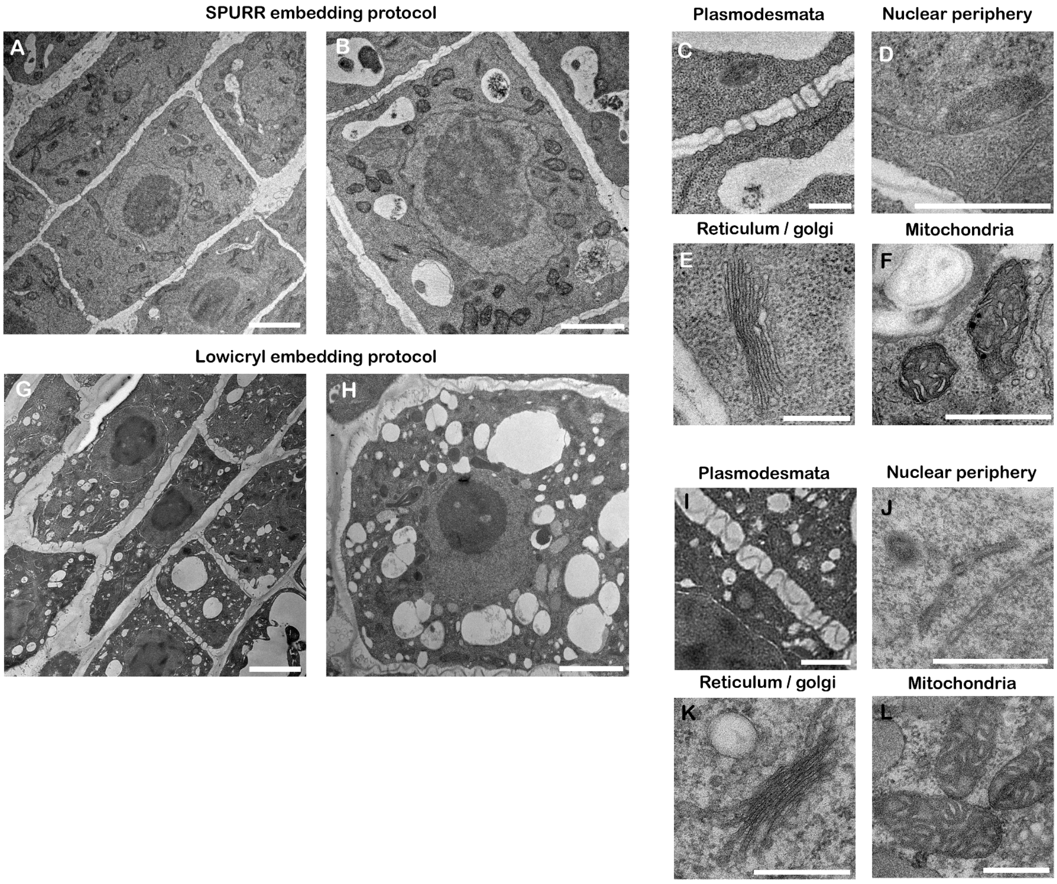

Supplementary Figure 1. Ultrastructural preservation of 70 nm tissue sections in Spurr's and Lowicryl embedding protocols

Ultrastructural integrity of Spurr's samples (A, B) and Lowicryl embedded samples (G, H). Application of the Spurr's protocol leads to a better contrasting of cellular structures (C – plasmodesmata; D –nuclear periphery) and organelles (E – reticulum / Golgi; F – mitochondria). Cellular structures remain recognizable in Lowicryl embedding protocol, with recognizable architecture of intracellular organelles (I – plasmodesmata; J – nuclear periphery; K – reticulum / Golgi; L – mitochondria). Legend: Nuc – nucleus, No – nucleolus, PDT – plasmodesmata, FC – fibrillar center, MT – mitochondria, NM – nuclear membrane, VS – vesicles. Scale bar (A. B. G. H): 2 µm; (C - F), (I - L): 500 nm.

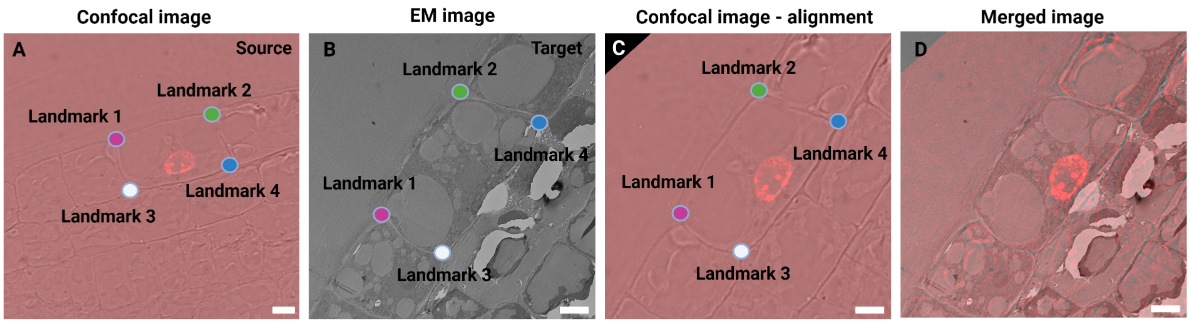

Supplementary Figure 2. Correlation of spinning disk and TEM micrographs using landmark features

Original spinning-disk confocal data (A) with the manual definition of landmarks, in this case, corners of the cell wall. EM image of the region of interest, with manual input of landmarks in the ec_CLEM Icy plugin (B). Alignment of the confocal image to the EM image (C), using the affine transformation and anisotropic noise models. Resulting overlay image of confocal and EM data (D). Landmarks are highlighted with colored dots. Scale bar: 5 µm.

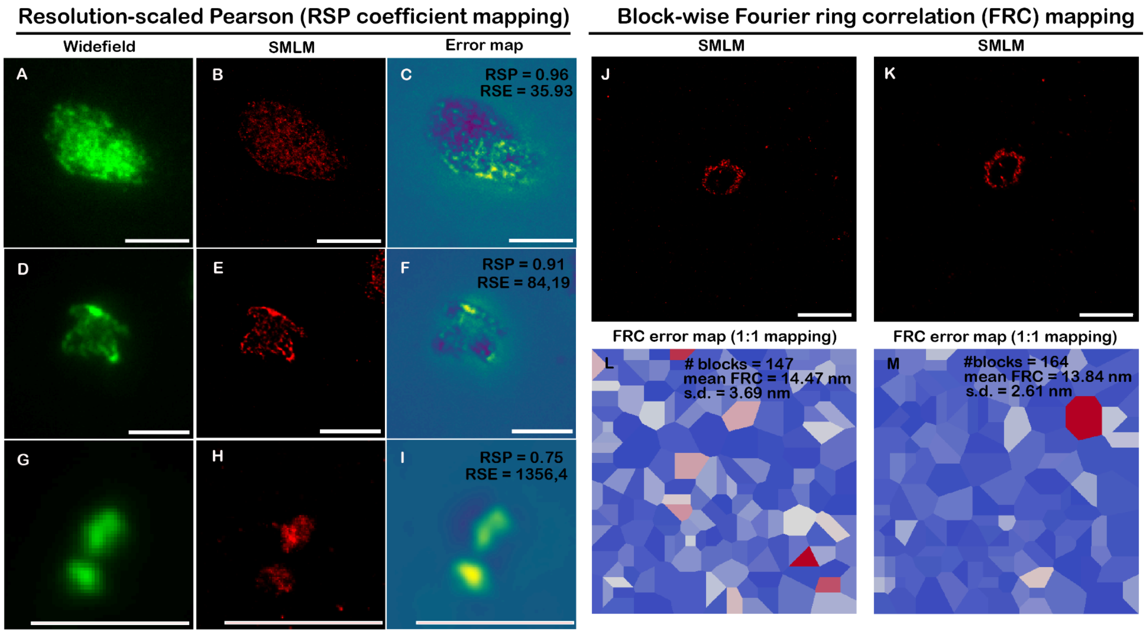

Supplementary Figure 3. Quantitative error mapping of SMLM reconstructions from sections in Lowicryl

Analysis of reconstruction errors using NanoJ-SQUIRREL. Global resolution-scaled Pearson coefficient (RSP) is calculated from the widefield (A, D, G) and reconstructed SMLM (B, E, H) images and provides an image quality metric (values ranging from -1 to 1, indicating full anti-correlation and correlation, respectively - C, F, I). Splitting of the original time series used for reconstruction (reconstruction in J, K) into different substacks can be used to calculate the Fourier ring correlation, used to estimate image resolution globally (mean FRC values), with local heterogeneities visible from the error maps (L, M). Scale bar: 5 µm.

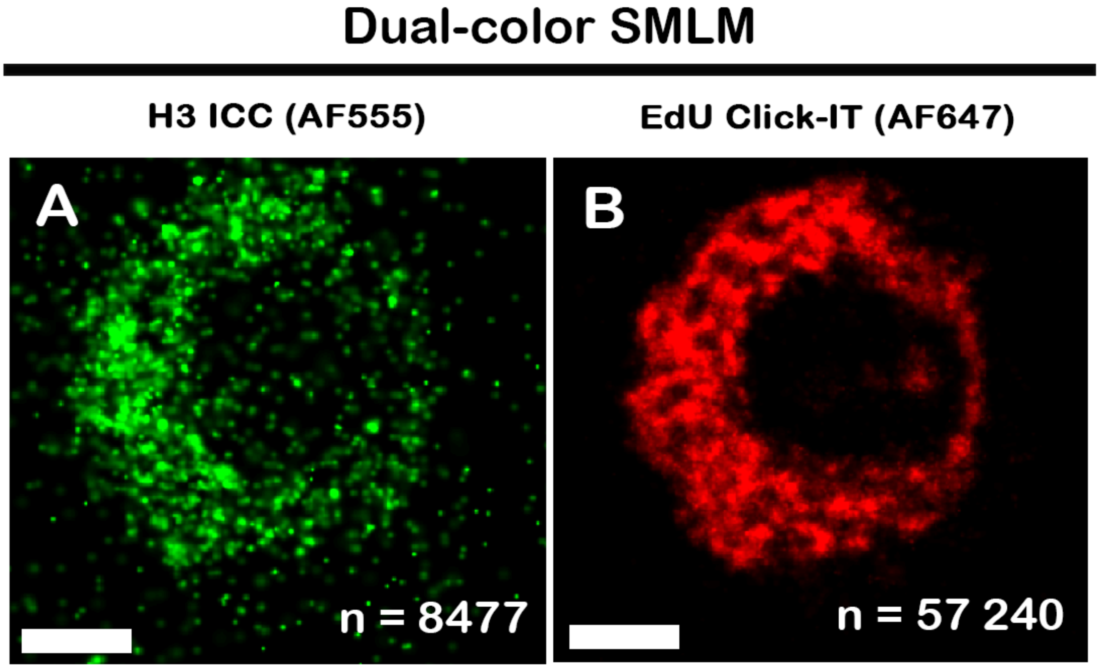

Supplementary Figure 4. Dual-color super-resolution microscopy on sections.

Antibody labelling of the H3 histone, with Alexa Fluor 555 secondary antibody (A). Click-iT labelling with EdU and Alexa Fluor 647 detection in (B). The number of localizations is indicated at the bottom left of each image, after filtering and image reconstruction in the ZEN Black software. ICC - immunocytochemistry. Scale bar: 1 µm.

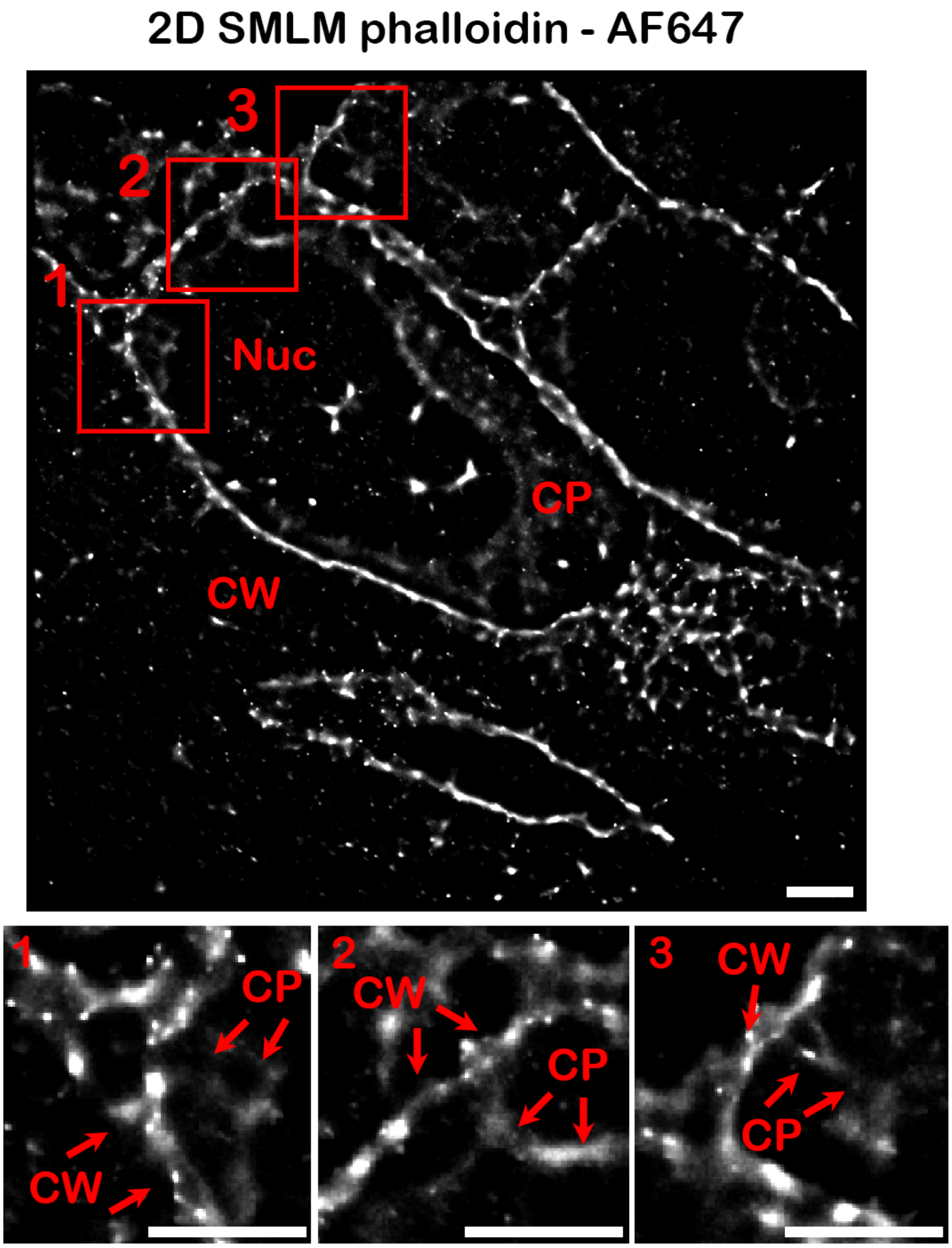

<u>**Supplementary Figure 5. Detection of actin ultrastructure in Lowicryl sections**</u>

SMLM analysis of actin in root tip cells. Insets (1-3) display actin branching from the cell wall fraction (CW) to the cytoplasmic structures (CP). CW - cell wall fraction, CP - cytoplasmic fraction. Scale bar: 2 µm.

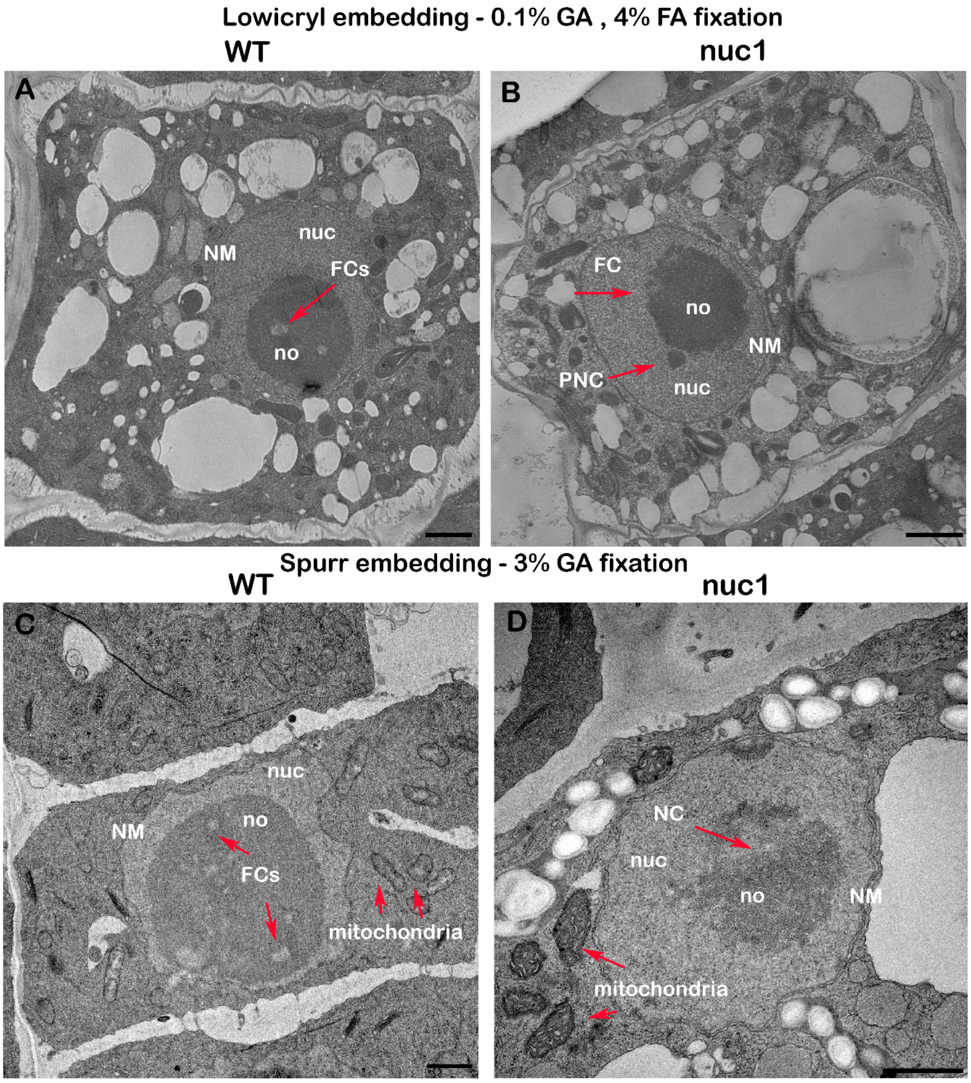

Supplementary Figure 6. Nucleolar ultrastructure in wild-type and *nucleolin 1* mutants

Differences in the nucleolar ultrastructure between wild-type (A, C) and *nuc1* (B, D) mutants in Lowicryl-embedded (A, B) and Spurr's-embedded (C, D) samples. Epidermal cells of wild-type plants show clearly delineated fibrillar centers (FCs, A, C). Some nucleoli of the *nucleolin 1* mutant show weak contrast in fibrillar centers with indistinct, diffuse edges (D). Nuc - nucleus, no - nucleolus, FC - fibrillar center. Scale bar (A, B): 2 µm, (C, D): 1 µm.
