# Supplementary material for: In-section Click-iT detection and super-resolution CLEM analysis of nucleolar ultrastructure and replication in plants
Source: Nat Commun. 2024 Mar 19;15:2445. doi: 10.1038/s41467-024-46324-6 (PMC10950858; doi:10.1038/s41467-024-46324-6)
Supplement: Supplementary file 4 — Description of Additional Supplementary Files [file 41467_2024_46324_MOESM4_ESM.pdf]

Description of Additional Supplementary Files

File Name: Supplementary Data 1

Description: contains a gallery of EM images as on overview of the ultrastructural preservation of the samples.
