# Supplementary material for: In-section Click-iT detection and super-resolution CLEM analysis of nucleolar ultrastructure and replication in plants
Source: Nat Commun. 2024 Mar 19;15:2445. doi: 10.1038/s41467-024-46324-6 (PMC10950858; doi:10.1038/s41467-024-46324-6)
Supplement: Supplementary file 5 — Supplementary Data 1 [file 41467_2024_46324_MOESM5_ESM.pdf]

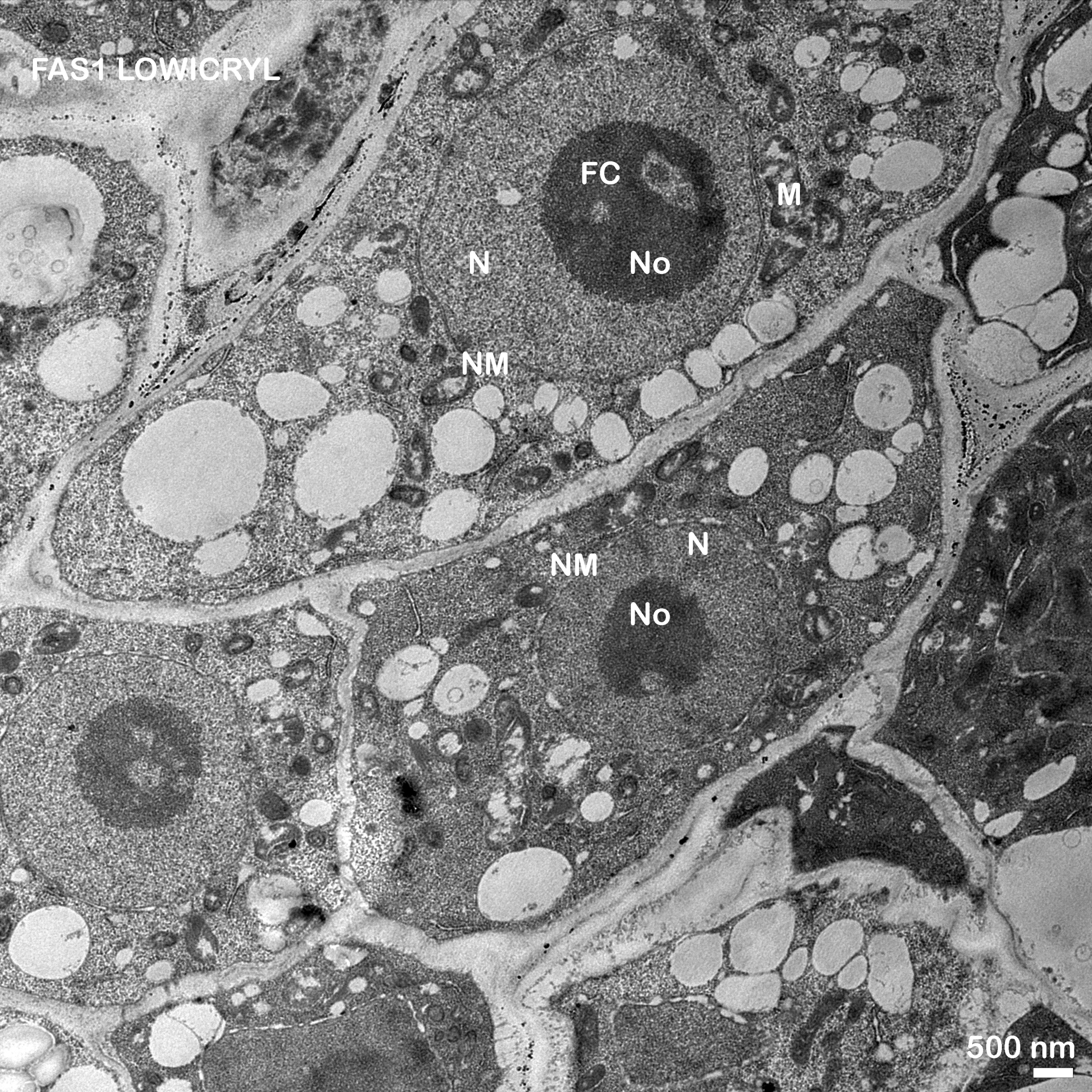
FAS1 LOWICRYL
FC
M
N
No
NM
NM
N
No
500 nm

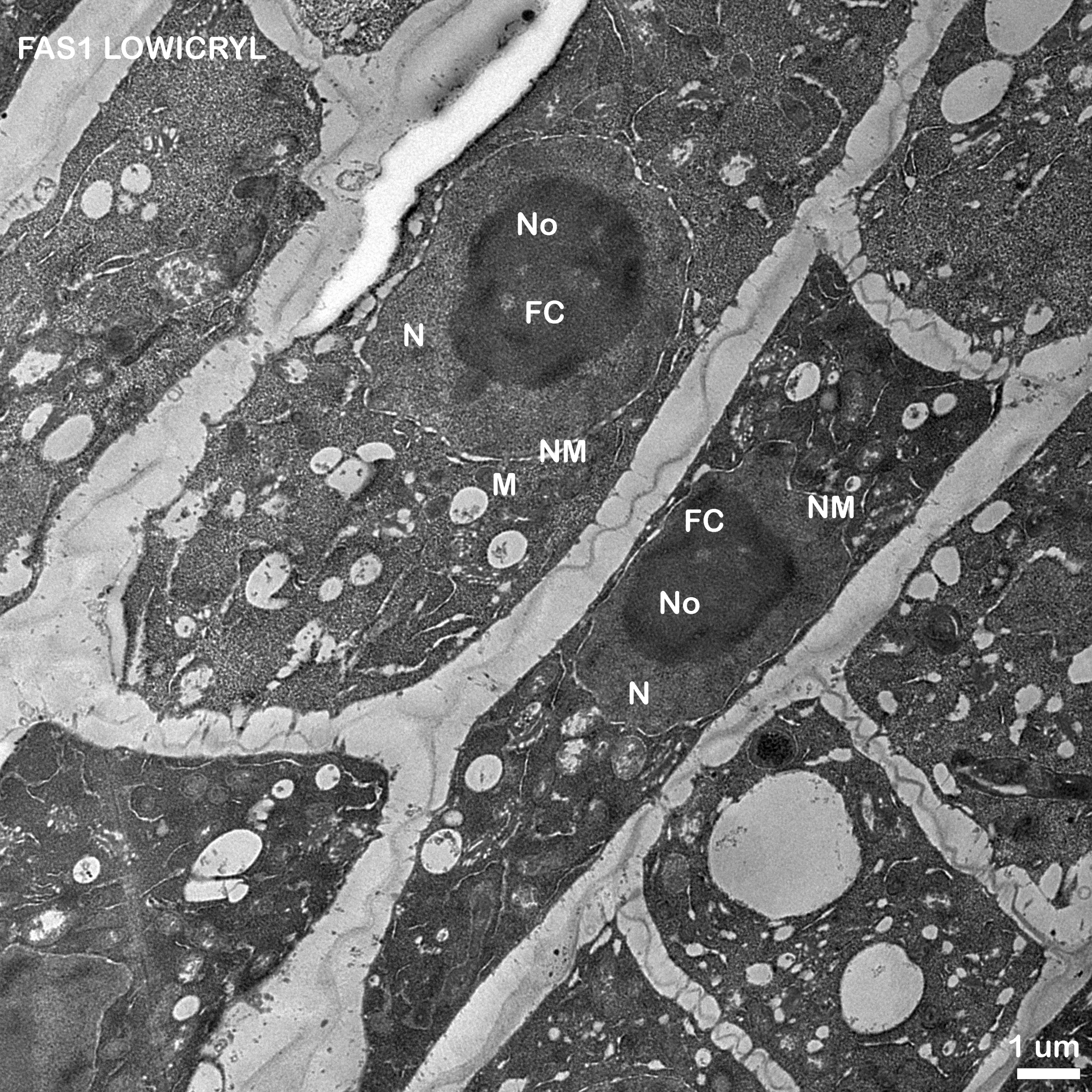
FAS1 LOWICRYL
No
FC
N
NM
M
FC
NM
No
N
1 um

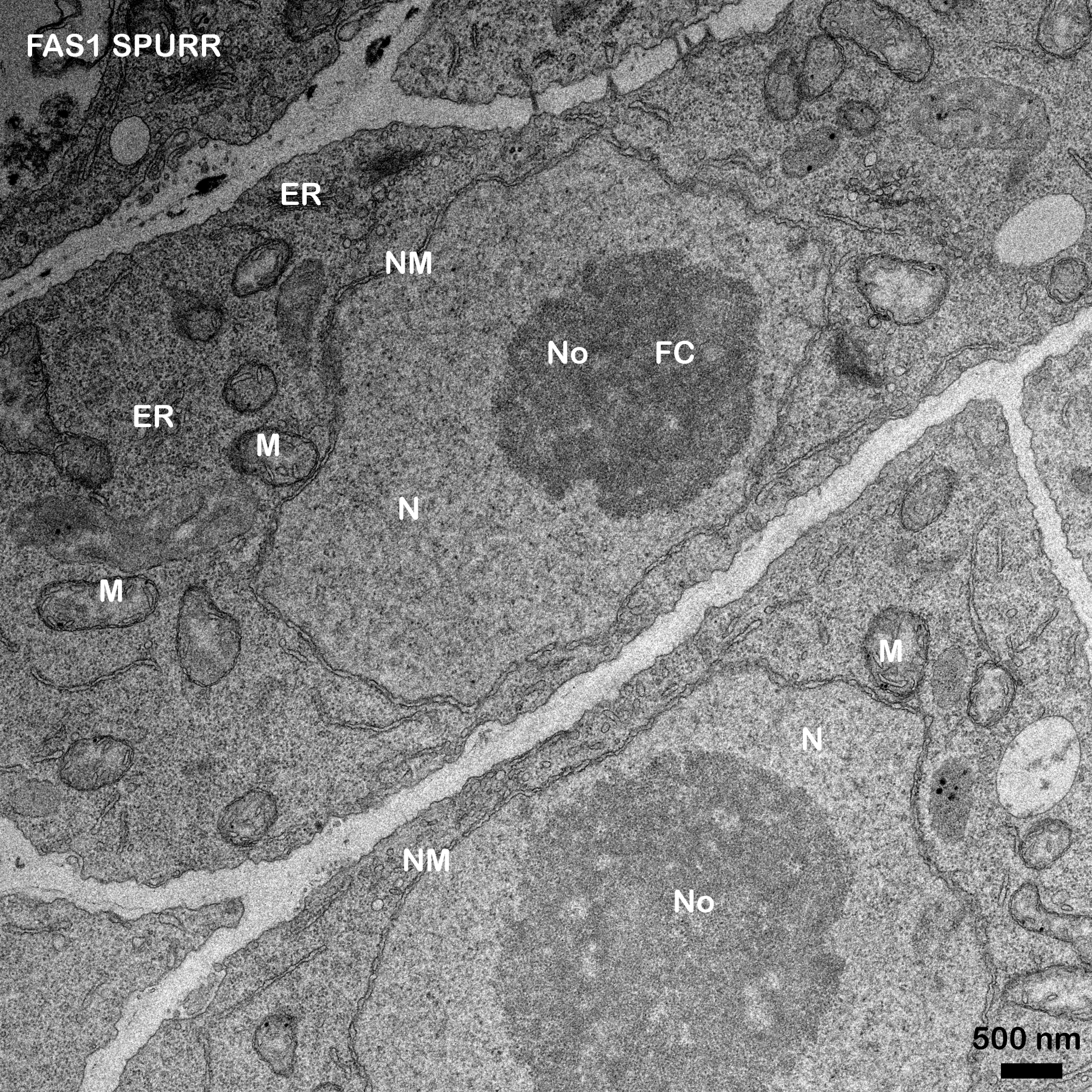
FAS1 SPURR
ER
NM
No
FC
ER
M
N
M
M
N
NM
No
500 nm

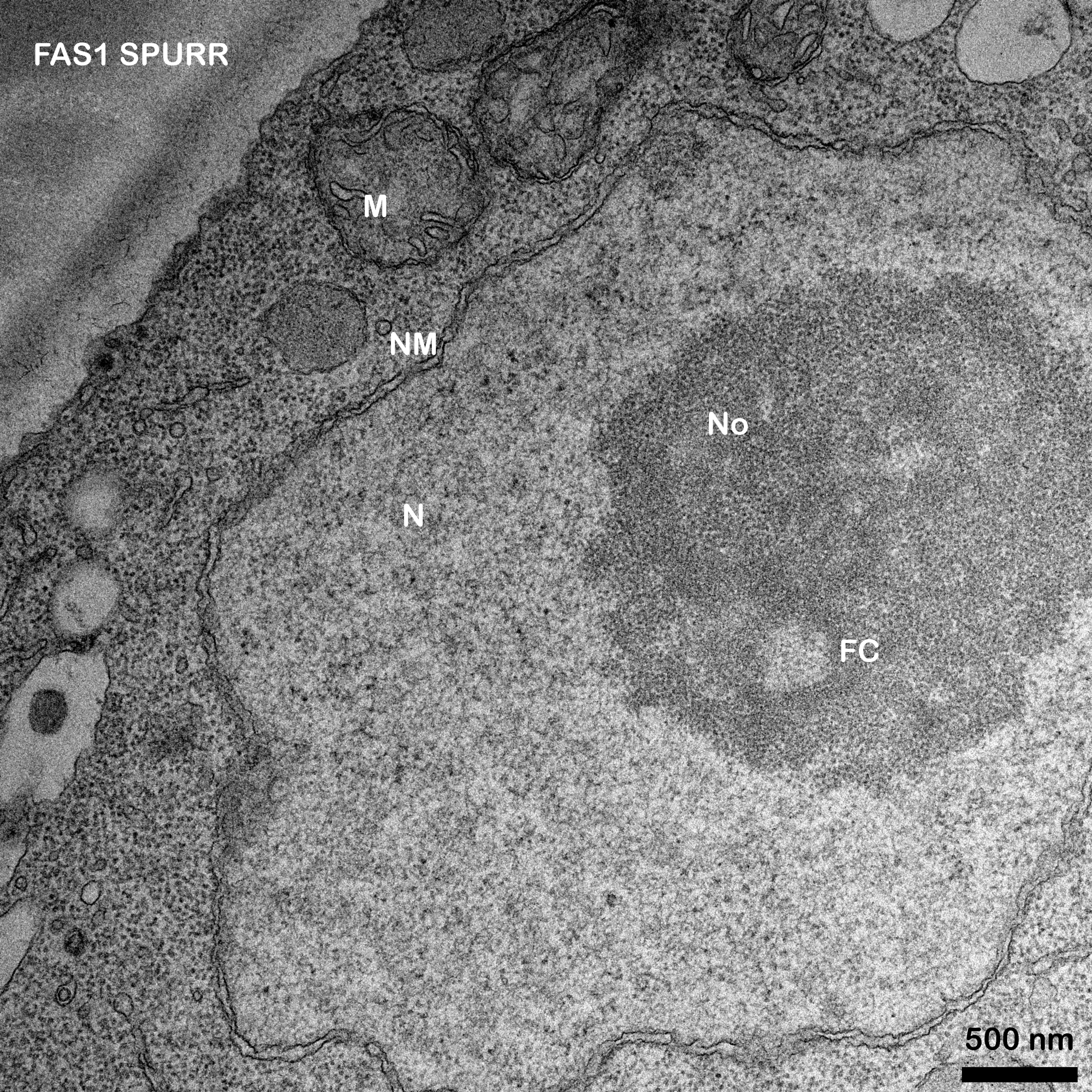
FAS1 SPURR
M
NM
No
N
FC
500 nm

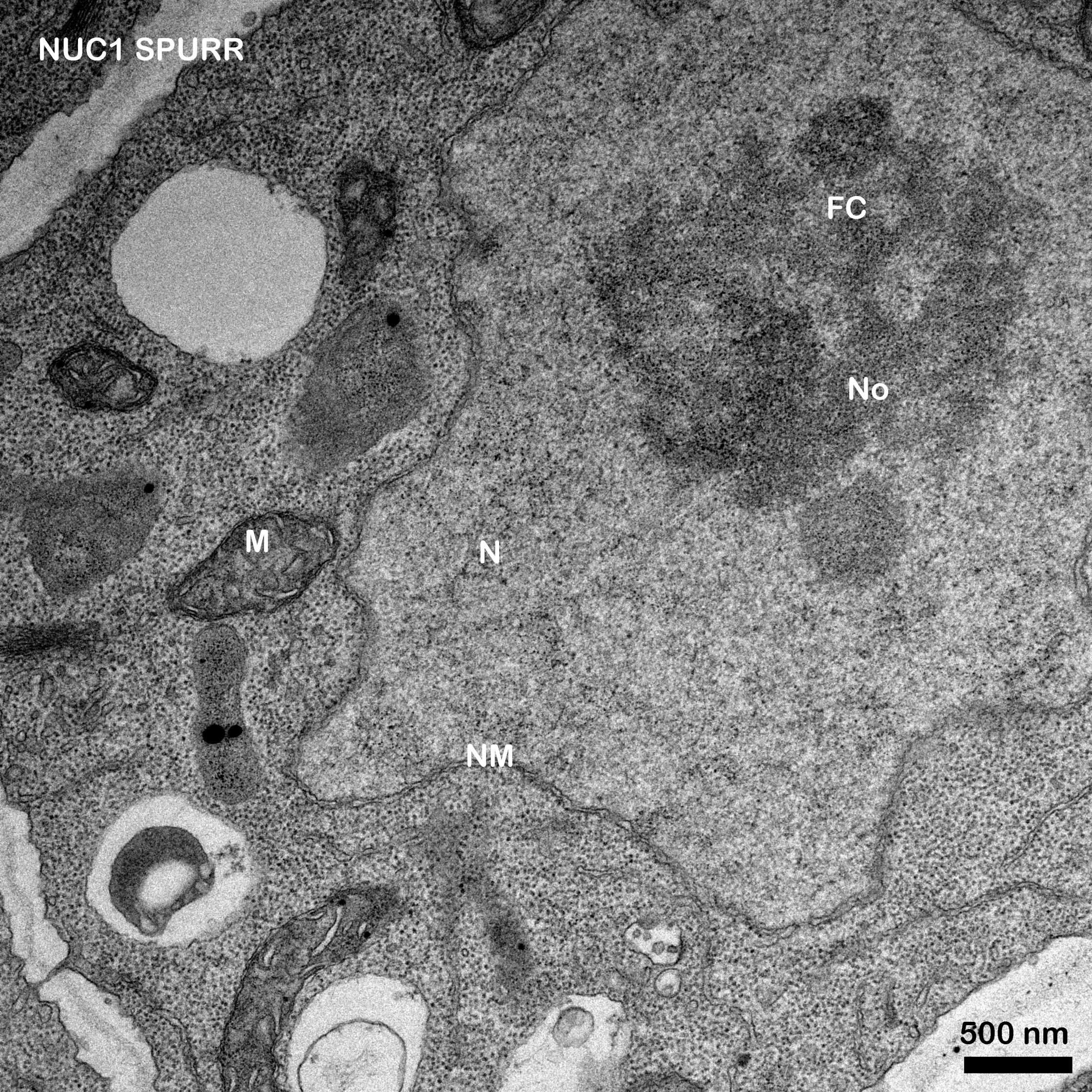
NUC1 SPURR
FC
No
M
N
NM
500 nm

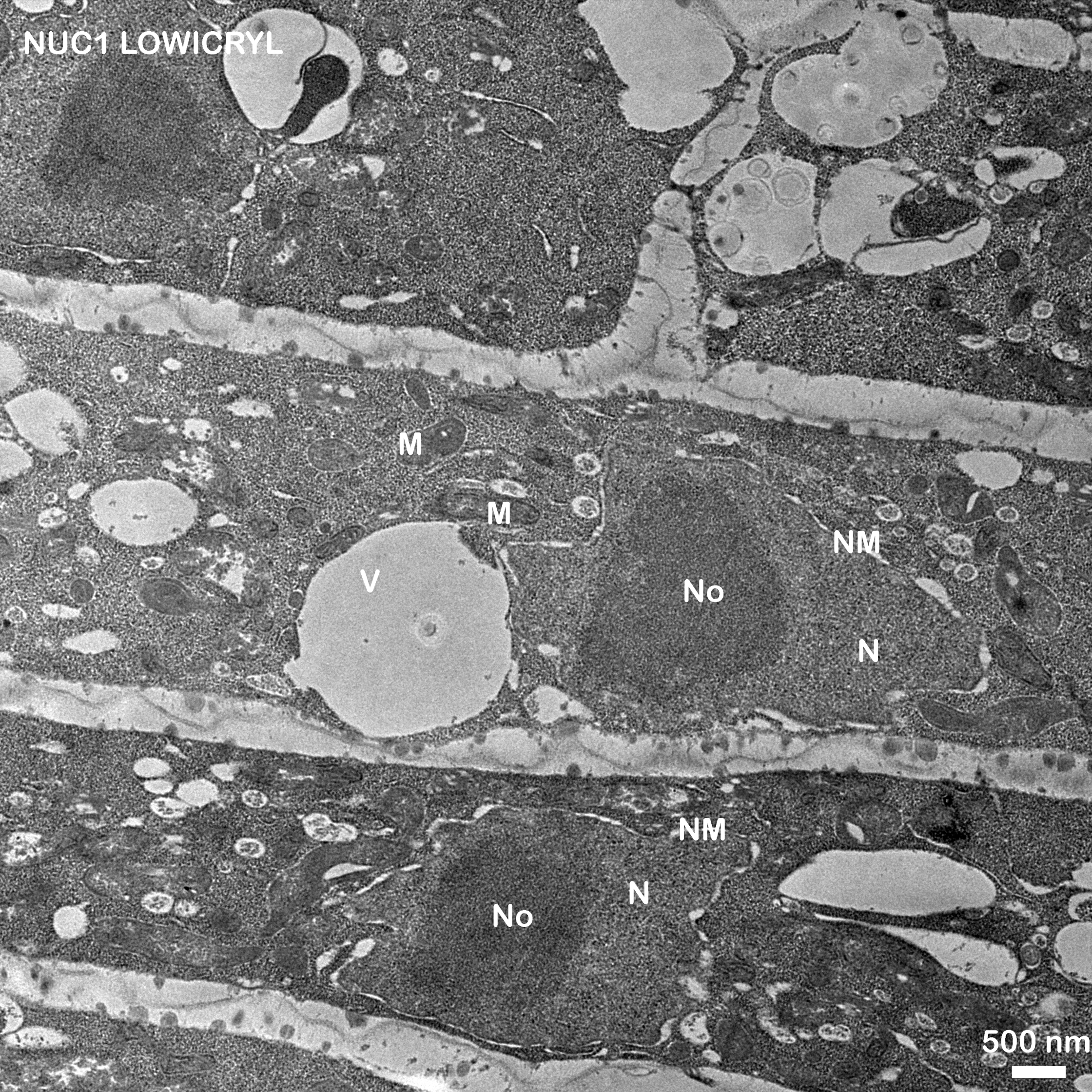
NUC1 LOWICRYL
M
M
V
No
NM
N
NM
N
No
500 nm

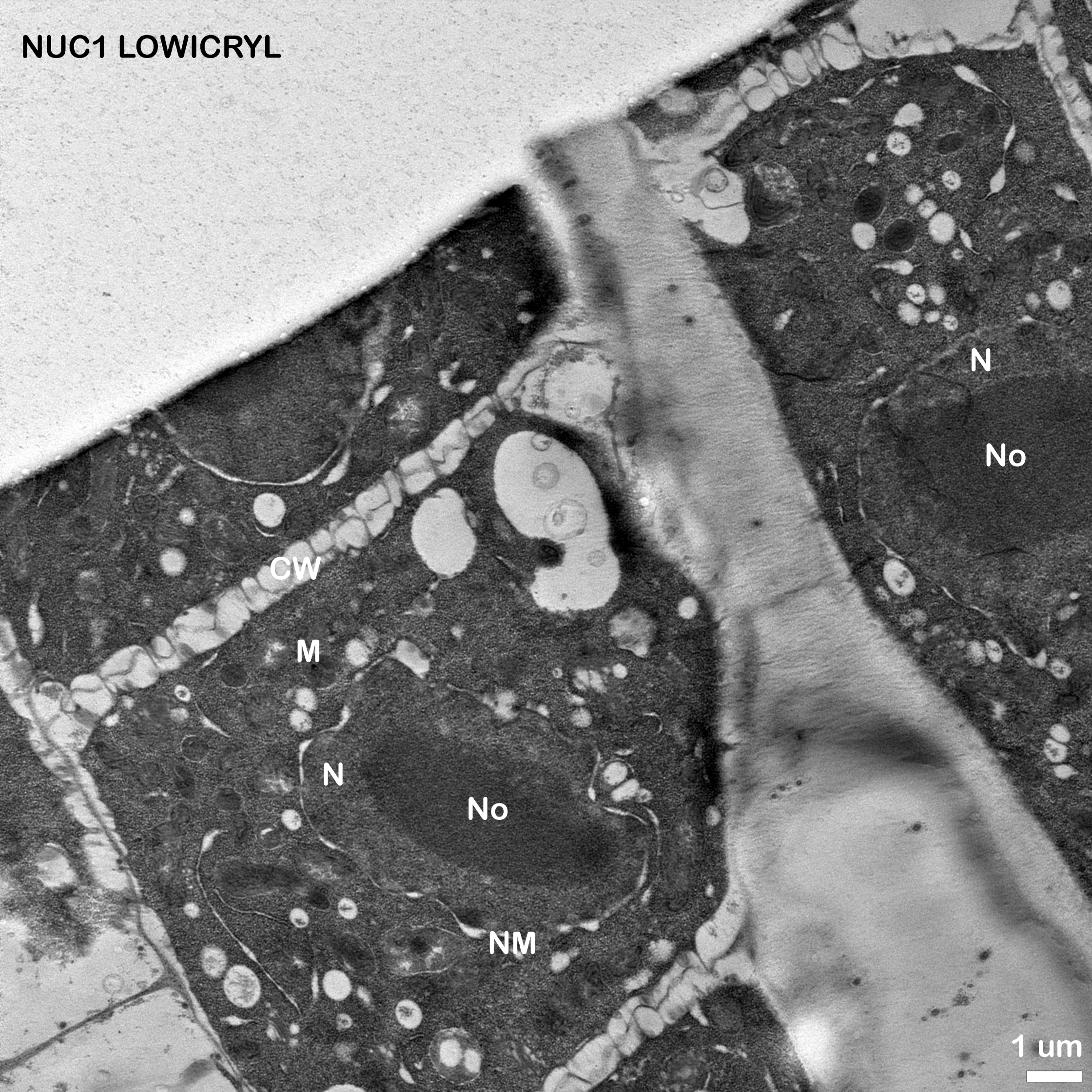
NUC1 LOWICRYL
N
No
CW
M
N
No
NM
1 um

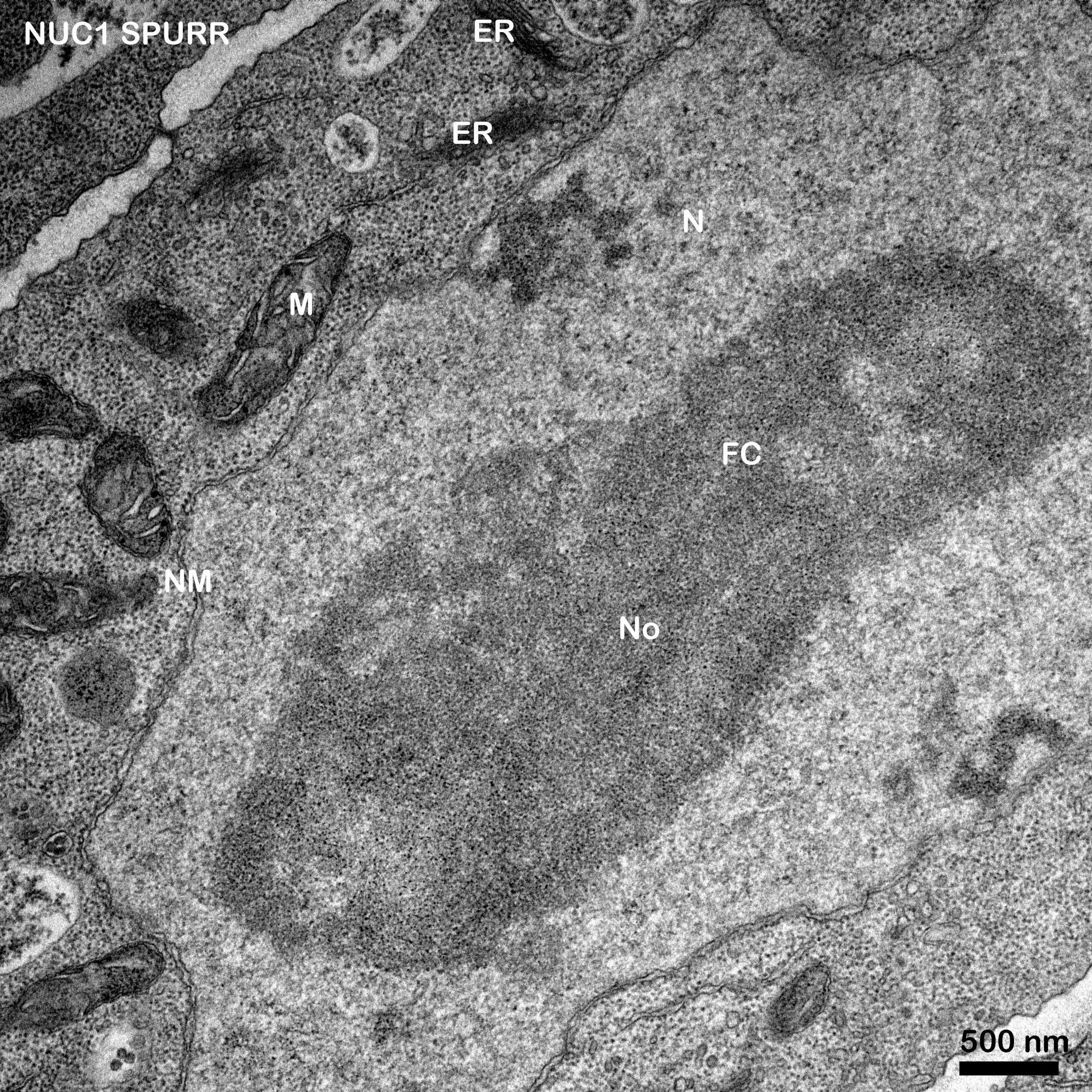
NUC1 SPURR
ER
ER
N
M
FC
NM
No
500 nm

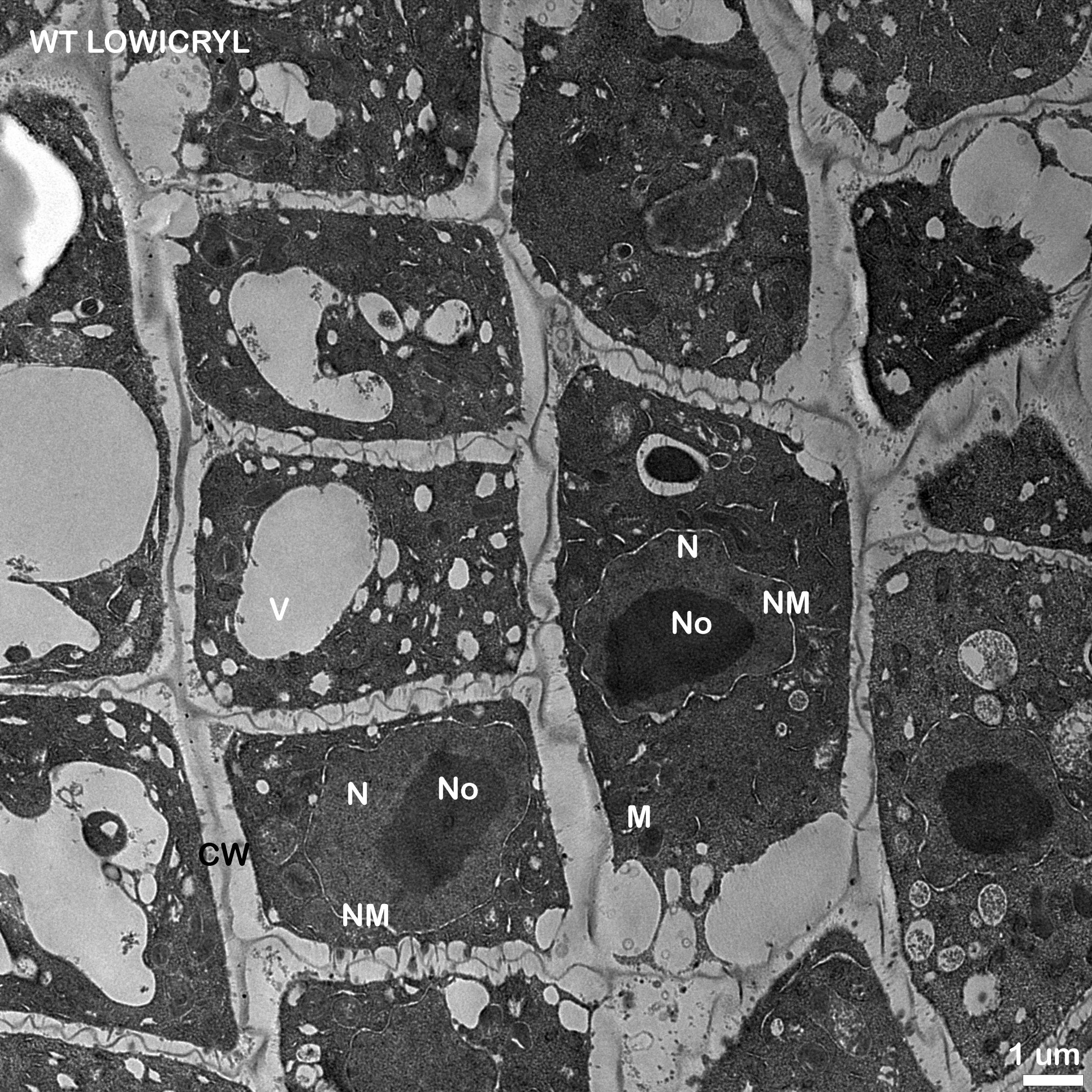
WT LOWICRYL
V
N
NM
No
N
No
M
CW
NM
1 um

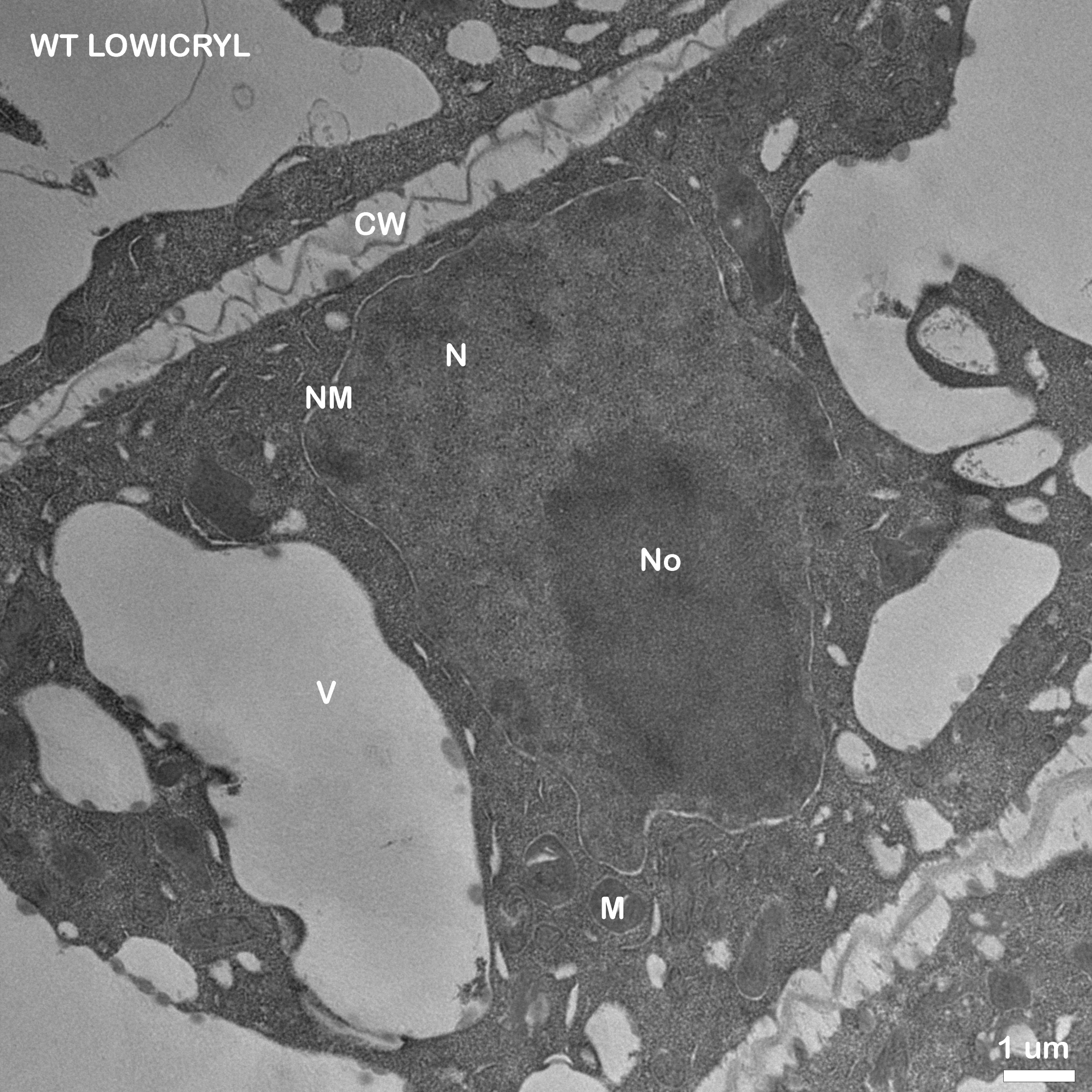
WT LOWICRYL
CW
N
NM
No
V
M
1 um

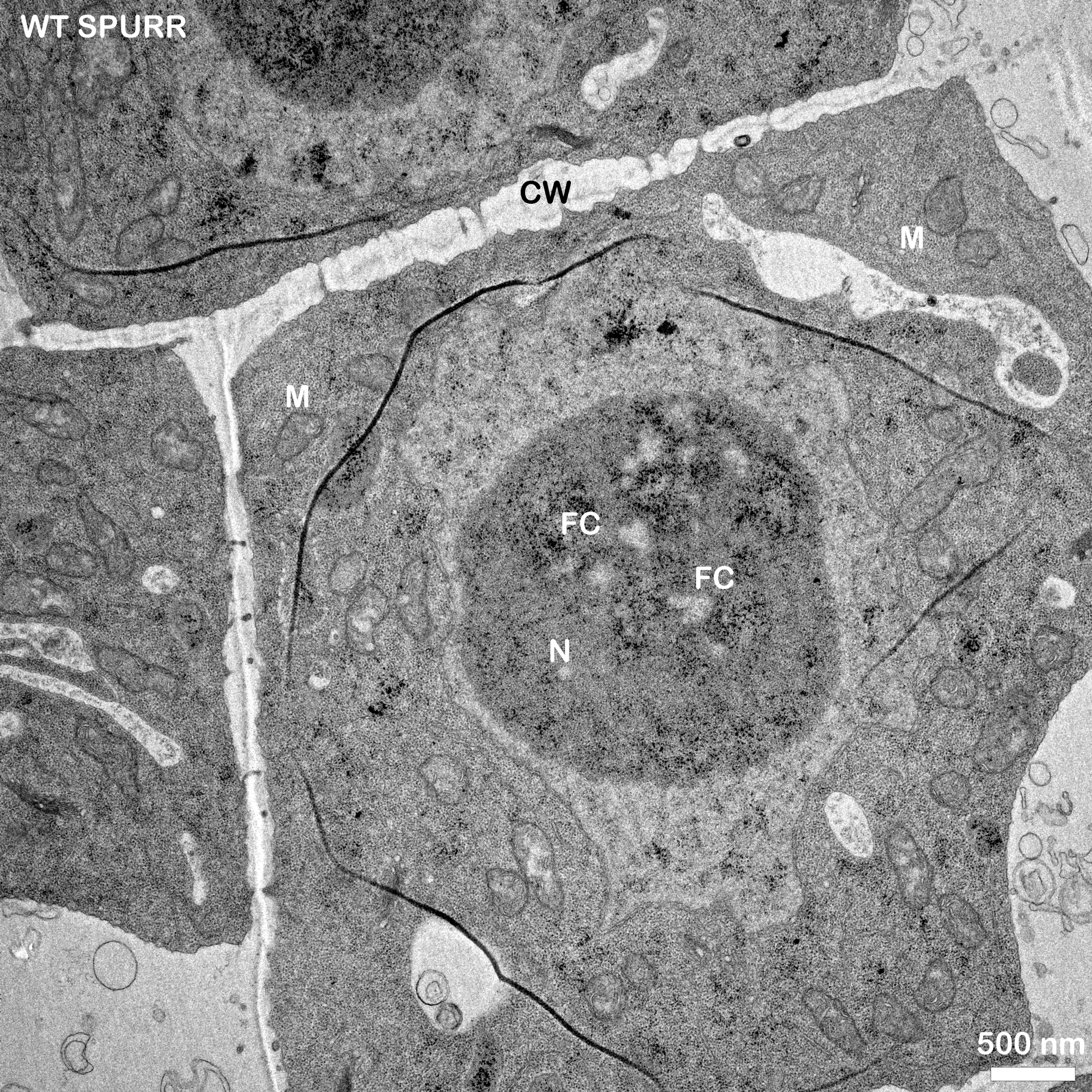
WT SPURR
CW
M
M
FC
FC
N
500 nm

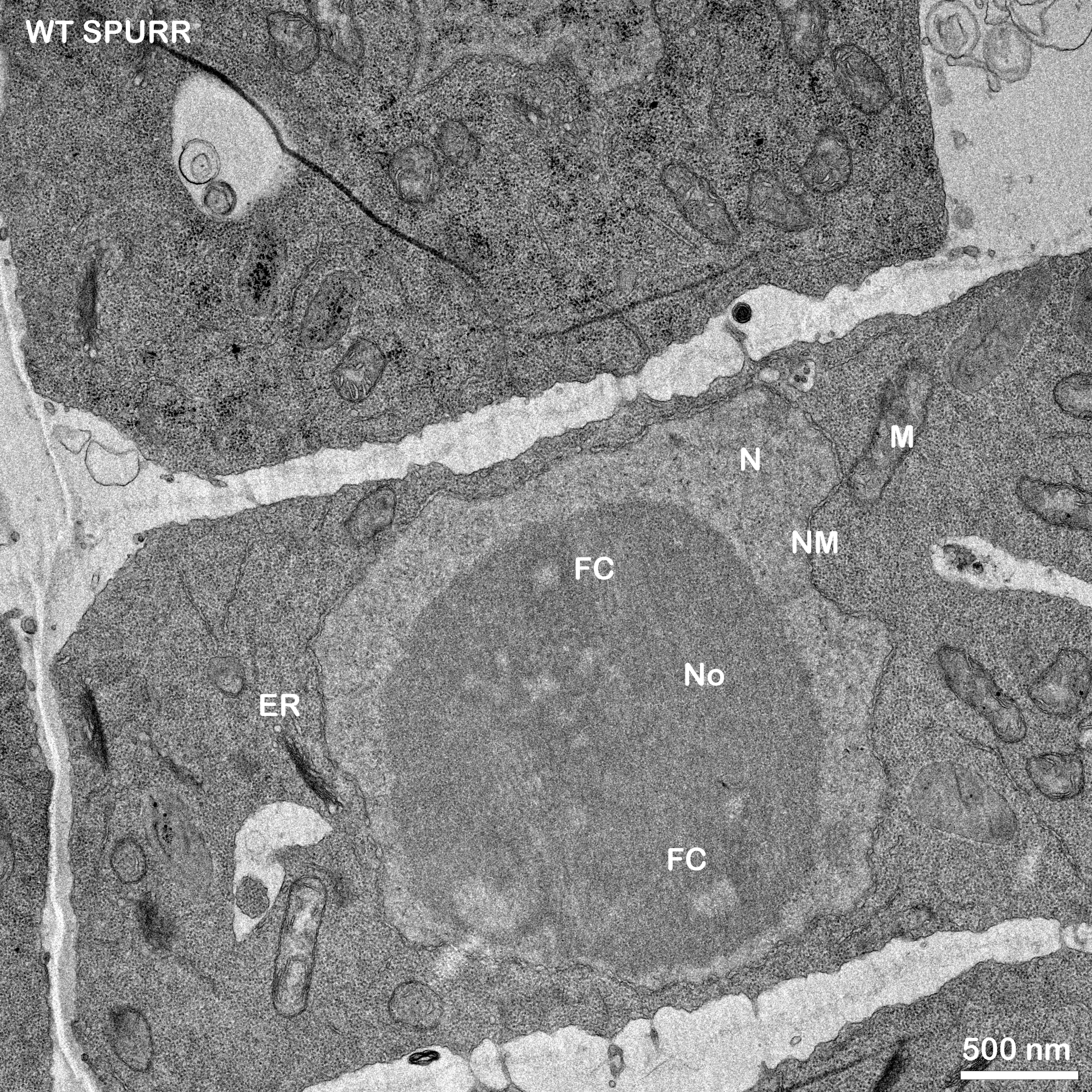
WT SPURR
M
N
NM
FC
No
ER
FC
500 nm
